# Mathematical Model of Stem Cell Differentiation and Tissue Regeneration with Stochastic Noise

**DOI:** 10.1007/s11538-014-9971-5

**Published:** 2014-07-18

**Authors:** Przemysław Rafał Paździorek

**Affiliations:** Institute of Mathematics of the Polish Academy of Sciences, Warsaw, Poland

**Keywords:** Stochasticity in the stem cells dynamics, Stochastic differential equations, Markov semigroups, Khasminskií function

## Abstract

Differentiation and self-renewal of stem cells is an essential process for the maintenance of tissue composition. The promise of novel medical therapies combined with the complexity of this process encourage us to employ numerical and mathematical methods. This will allow us to understand better the mechanisms which regulate stem cell behaviour. Perturbations to the cellular environment may have an influence on the death rate, proliferation rate and on the fraction of self-renewal at every stage of differentiation. In this paper, we present mathematical study of the effect of stochastic noise on the process of tissue regeneration. Here, a system of Itô stochastic differential equations with linear diffusion coefficients that is based on a deterministic model of multistage cell lineages is investigated. Numerical simulations of the stochastic model are shown for a different number of stages of differentiation. Interactions between the noise, added to the different stages, are characterised using numerical simulations. The long-time behaviour of the two-dimensional version of the model is fully characterised; asymptotic stability of the related Markov semigroup is proved using the theory of the Markov semigroups and the method of the Khasminskií function.

## Introduction

Tissues are composed of a large number of cells with different functions and morphology. However, most of mature cells are not able to proliferate. Instead, mature cells are replenished by the presence of population of adult stem cells which possess the ability to self renew and to differentiate into various cell lineages. The key role of stem cells is to supply tissue with new cells in homeostatic manner.

The process of maintaining the number of mature cells is a well regulated, multistep process, consisting of differentiation of stem cells and their progeny and of the self-renewal of the stem cell population. The process of differentiation may be divided into several stages. Both stem cells and their progeny can self-renew (Marciniak-Czochra et al. 2009). However, when the progeny of the stem cells enter to the higher stages of differentiation, their ability to differentiate is reduced and ultimately lost when they reach full maturity.

Mechanisms of regulation of these complex processes are not well understood. The promise of novel stem cells-based therapies, such as therapies for impaired organs, degenerative diseases (Ehnert et al. 2009; Gratwohl and Baldomero 2009; Macarthur et al. 2009) or reconstitution of blood structure after chemotherapy in treatment of leukemias (Marciniak-Czochra and Stiehl 2012), has led to increase of interest in this field. Several mathematical and numerical models were developed to help in understanding stem cell differentiation (Stiehl and Marciniak-Czochra 2010; Marciniak-Czochra et al. 2009; Till et al. 1964; Wu et al. 2009; Colijin and Mackey 2005).

The aforementioned models present different mathematical approaches to describe processes of differentiation and self renewal (Stiehl and Marciniak-Czochra 2010; Marciniak-Czochra et al. 2009; Doumic et al. 2011) involving stem cells proliferation (Till et al. 1964) and mechanisms of stem cell fate decision (Wu et al. 2009) or stem cells regulation system (Colijin and Mackey 2005). Each of these models describes different parts of the process of homeostasis in adult tissue. However, an important difference is how an environmental and intracellular perturbations during those processes are considered, and if they have a reflection in the mathematical form of the models.

The main purpose of this study is to investigate a stochastic stability of the system presented in Marciniak-Czochra et al. (2009) and study how the model responds to noise. We choose this particular model, for its simplicity and for its general macroscopic approach to the problem. An additional advantage of this model is a promising application in describing blood reconstitution after chemotherapy (Marciniak-Czochra and Stiehl 2012).

The paper is organised as follows. First, we present a short description of the model developed in Marciniak-Czochra et al. (2009) and also the biological and mathematical assumptions related to this model. Then, we propose a stochastic version of this model using a system of stochastic ordinary differential equations that are in the form of the original model. Further, we present some numerical simulations of the stochastic model. In the Appendix, we prove mathematical proof of the long-time behaviour of the distribution of the stochastic process described by the stochastic version of the model. We study the evolution of the distribution of the solution of stochastic version of the model which is related to some Markov semigroup. We also investigate the existence of the stationary distribution of the stochastic model. Finally, the asymptotic stability of the Markov semigroup related to the stochastic model is shown. The proof is based on the theory of Markov semigroups and the method of the Khasminskií function (Rudnicki et al. 2002; Pichór and Rudnicki 1997; Skwara 2010a, b).

## Deterministic Model

This section is devoted to the deterministic model proposed in Marciniak-Czochra et al. (2009) and analysed in Stiehl and Marciniak-Czochra (2010), Nakata et al. (2012). We assume that there exists a discrete chain of differentiation stages and skipping of some stages during the process of differentiation is impossible. Cell behaviour at each stage of maturation is described by parameters of death rate, proliferation rate and a probability of differentiation. Additionally, it is assumed that the system is regulated by a single cytokine similarly as the red blood cells production is controlled by erythropoietin (Fried 2009; Metcalf 2008; Lasota et al. 1981; Adamson 1994; Ratajczak et al. 1997) or the process of specializing of granulocytes is by G-CSF (Metcalf 2008; Semerad et al. 2002; Price et al. 1996).

Let $c_1,\dots , c_n$ describe the density of population of stem cells, cells at $i$th stage of differentiation and mature cells, respectively. According to the papers (Stiehl and Marciniak-Czochra 2010; Marciniak-Czochra et al. 2009; Marciniak-Czochra and Stiehl 2012) the phrase ‘density of the population’ corresponds to the size of the population. Let $s$ be the concentration of the signalling molecules. Following Marciniak-Czochra et al. (2009) it is assumed that the concentration $s$ depends only on the density of mature cells $c_n$. Assuming that the dynamics of the cytokine is a fast process and

1 $$\begin{aligned} \frac{\mathrm{{d}}s}{\mathrm{{d}}t}=\mu (1-s-ksc_n(t)), \end{aligned}$$

where $\mu$ is the death rate of the mature cells population, and $k$ is a positive constant. Using a quasi-steady state approximation yields to an algebraic dependence of the form described by

2 $$\begin{aligned} s_k(t)=\frac{1}{1+kc_n(t)}. \end{aligned}$$

Assuming that both proliferation and differentiation processes are regulated leads to the following system of differential equation describing dynamics of $n$ cell subpopulations

3 $$\begin{aligned} \frac{\mathrm{{d}}c_1}{\mathrm{{d}}t}&= (2a_1s_{k_1}(t)-1)p_{1}s_{k_2}(t)c_1(t)-\mu _1 c_1,\nonumber \\ \frac{\mathrm{{d}}c_2}{\mathrm{{d}}t}&= (2a_2s_{k_1}(t)-1)p_{2}s_{k_2}(t)c_2(t)+2(1-a_1s_{k_1} (t))p_{1}s_{k_2}(t)c_1(t)-\mu _2c_2(t), \nonumber \\&\quad \vdots \nonumber \\ \frac{\mathrm{{d}}c_n}{\mathrm{{d}}t}&= 2(1-a_{n-1}s_{k_1}(t))p_{n-1}s_{k_2}(t)c_{n-1}(t)-\mu _n c_n(t), \end{aligned}$$

where $p_i$ for $i=1,\dots , n-1$ is the proliferation rate of the population at stage $i$, $\mu _i$ for $i=1,\dots , n$ denotes the death rate at the stage $i$ and $a_i$ for $i=1,\ldots , n-1$ is the maximal fraction of self-renewal at the stage $i$. The real fraction of self-renewal at time $t$ is then given by $a_is_{k_1}(t)$ and defined as a fraction of the direct progeny of cells at stage $i$ which are at the same differentiation stage as their progenitors. The following assumptions are made

4 $$\begin{aligned} t&\in [0, \infty ), \nonumber \\ c_{i,0}&\ge 0 \quad \mathrm{for} i=1\ldots n, \nonumber \\ \mu _i&\ge 0 \quad \mathrm{for} i=1\ldots n-1, \nonumber \\ \mu _n&> 0,\nonumber \\ p_i&\ge 0 \quad \mathrm{for} i=1\ldots n, \nonumber \\ a_i&\in [0,1] \quad \mathrm{for} i=1\ldots n. \end{aligned}$$

The death rate, proliferation rate and initial conditions are non-negative and the fraction of self-renewal is between $0$ and $1$, which corresponds to different types of differentiation: symmetric self-renewal, symmetric differentiation and asymmetric divisions.

The model is based on the assumption that cells divide at the rate $p_is_{k2}(t)$, which results into $p_is_{k2}c_i(t)$ of descendant cells in a unit time $t$ and stage $i=1,\dots ,n$. The fraction $a_i$ of progeny cells remains at the same stage of differentiation as the parent cell, while $1-a_i$ fraction of the progeny cells differentiates, i.e. transfers to the higher differentiation stage. Additionally, cell death at the rate $\mu _i$ is modelled.

We also note that when the population of mature cells $c_n$ reaches some value, then the term $(2a_is_{k_1}(t)-1)p_{i}s_{k_2}(t)c_i(t)$ becomes negative and the number of cells at stage $i$ decreases. On the other hand when the density of the mature cells is low, then $(2a_is_{k_1}(t)-1)p_{i}s_{k_2}(t)c_i(t)$ is positive and the number of cells at stage $i$ increases providing that the death rates are not too high. It shows how the dynamics of each cell subpopulation depends on the level of mature cells.

The model (3) is well posed, the solution exists and it is unique for $t\in [0,\infty )$ and for non-negative initial condition, the solution of system (3) remains non-negative, which is proved in Stiehl and Marciniak-Czochra (2010). Additionally, assuming that

5 $$\begin{aligned} d_1&< (2a_1-1)p_1, \nonumber \\ 0&< 2a_1p_1(d_i+p_i)-2a_ip_i(d_1+p_1), \quad \mathrm{for} \;\; i=2,\dots ,n-1. \end{aligned}$$

it is shown that system (3) has a unique positive steady-state (Stiehl and Marciniak-Czochra 2010). The first inequality provides that there exists a level of the density of mature cells such that $c_1'>0$. In other words, the population of stem cells does not simply decrease and becomes extinct but replenishes itself. The second inequality of (5) says that the signal intensity for self-maintenance of the population of stem cells is lower than the concentration needed at some stage of maturation $i$ to maintain the population without influx from the stage $i-1$. This interpretation is straightforward if we set $d_1=\cdots =d_{n-1}=0$ and $d_n>0$. Then, we obtain that condition (5) is equivalent to

6 $$\begin{aligned} a_1&> \frac{1}{2},\nonumber \\ a_1&> a_i, \quad \mathrm{for}\;\; i=2,\ldots ,n-1. \end{aligned}$$

The latter assumption on the death rates might be applied to cell systems, such as granulopoietic system (Stiehl and Marciniak-Czochra 2010).

In previous works, different numbers of compartments $n$ were chosen. As proposed in Marciniak-Czochra and Stiehl (2012) $n=8$ is a direct reflection of the biological case of differentiation of white blood stem cells into Neutrophil Granulocyte (Jandl 1996). The number $n=6$ considered in Marciniak-Czochra et al. (2009) corresponds to long term repopulating stem cells, short term repopulating stem cells, multipotent progenitor cells, committed progenitor cells, precursors and mature cells. We may also consider model simplifications with $n=2$ or 3. In the first case we treat all immature cells jointly with stem cells as one population and mature cells as another one. The second case is related to the properties of dividing cells. The first compartment might be understood as stem cells and their direct progeny, which are characterised by a low frequency of divisions, the second one as the direct progenitors of mature cells, which are characterised by a high frequency of division. The third compartment is treated as a population of mature cells, which do not divide any more.

## Stochastic Model

In this chapter, we present a stochastic version of the deterministic model (3). We convert the latter deterministic system of differential equations to a system of Itô stochastic differential equation in the following way:

7 $$\begin{aligned} \mathrm{{d}}\xi _1&= \left( \left( \frac{2a_1}{1+k\xi _n}-1\right) p_1\xi _1 -\mu _1 \xi _1\right) \mathrm{{d}}t+\alpha _1\xi _1\mathrm{{d}}W_{1,t}, \nonumber \\ \mathrm{{d}}\xi _2&= \left( \left( \frac{2a_2}{1+k\xi _n}-1\right) p_2\xi _2+2\left( 1-\frac{a_1}{1+k\xi _n}\right) p\xi _1-\mu _2\xi _2\right) \mathrm{{d}}t +\alpha _2\xi _2\mathrm{{d}}W_{2,t},\nonumber \\&\quad \vdots \nonumber \\ \mathrm{{d}}\xi _n&= \left( 2\left( 1-\frac{a_{n-1}}{1+k\xi _n}\right) p_{n-1}\xi _{n-1}-\mu _n \xi _n\right) \mathrm{{d}}t+\alpha _n\xi _n\mathrm{{d}}W_{n,t}, \end{aligned}$$

where stochastic processes $\xi _1$, $\xi _2$, $\dots$, $\xi _n$ describe the same variables as in the model (3), the densities of the population of stem cells, cells at different stages of differentiation and mature cells. Coefficients $a_i$, $p_i$ for $i=1,\dots ,n-1$ and $\mu _i$ for $i=1,\dots n$ satisfy assumptions (4), (5). $(W_{1,t},\dots W_{n,t})$ is an $n$-dimensional Wiener process and $\alpha _i$ for $i=1,\dots ,n$ are positive.

In the rest of this chapter we will consider the following $2$-dimensional version of the model (7):

8 $$\begin{aligned} \mathrm{{d}}\xi _1&= \left( \frac{2a}{1+k\xi _2}-1\right) p\xi _1\mathrm{{d}}t+\alpha _1\xi _1\mathrm{{d}}W_{1,t}, \nonumber \\ \mathrm{{d}}\xi _2&= \left( 2\left( 1-\frac{a}{1+k\xi _2}\right) p\xi _1-\mu \xi _2\right) \mathrm{{d}}t+\alpha _2\xi _2\mathrm{{d}}W_{2,t}, \end{aligned}$$

where as was mentioned in the previous section, processes $\xi _1,$ may be interpreted as a density of population of cells which are not yet differentiated and $\xi _2$ as a density of mature cells population. The latter model is well posed, the solution exists and is unique, which results directly from the following consideration. System (8) is understood as a solution of the following stochastic integral equation

9 $$\begin{aligned} \xi _1(t)&= \xi _1(0)+\int \limits _0^t\left( \frac{2a}{1+k\xi _2}-1\right) p\xi _1\mathrm{{d}}t+\int \limits _0^t\alpha _1\xi _1\mathrm{{d}}W_{1,t}, \nonumber \\ \xi _2(t)&= \xi _2(0)+\int \limits _0^t\left( 2\left( 1-\frac{a}{1+k\xi _2}\right) p\xi _1-\mu \xi _2\right) \mathrm{{d}}t+\int \limits _0^t\alpha _2\xi _2\mathrm{{d}}W_{2,t}. \end{aligned}$$

One may note that the following formulas are bounded

10 $$\begin{aligned} -1&\le \frac{2a}{1+k\xi _2}-1\le 2a-1 \nonumber \\ 0&\le 2\left( 1-\frac{a}{1+k\xi _2}\right) p \le 2p. \end{aligned}$$

Using comparison theorem for one-dimensional Itó process ((Ikeda and Watanabe 1981, p. 352)) and (10) we may find two stochastic processes $(N_1(t),N_2(t))$, $(M_1(t),M_2(t))$ described by the following systems of Itô stochastic differential equations

11 $$\begin{aligned} \mathrm{{d}}M_1&= -pM_1\mathrm{{d}}t+\alpha _1M_1\mathrm{{d}}W_{1,t} \nonumber \\ \mathrm{{d}}M_2&= -\mu M_2\mathrm{{d}}t+\alpha _2M_2\mathrm{{d}}W_{2,t} \end{aligned}$$

12 $$\begin{aligned} \mathrm{{d}}N_1&= (2a-1)pN_1\mathrm{{d}}t+\alpha _1N_1\mathrm{{d}}W_{1,t}, \nonumber \\ \mathrm{{d}}N_2&= (2pN_1-\mu N_2)\mathrm{{d}}t+\alpha _2N_2\mathrm{{d}}W_{2,t}, \end{aligned}$$

and satisfying the following inequalities

13 $$\begin{aligned} M_1(t)&\le \xi _1(t) \le N_1(t), \nonumber \\ M_2(t)&\le \xi _2(t) \le N_2(t) \quad \mathrm a.s. \end{aligned}$$

Since a solution of the system (11)–(12) exists and is unique, then the process described in Eq. (9) exists and is unique. It is straightforward that the processes $\xi _1$, $\xi _2$ are non-negative if and only if the initial processes are non-negative. Since the solution of the Eqs. (11) is as follows

14 $$\begin{aligned} M_1(t)&= M_1(0)\exp \left\{ \left( -p-\frac{\alpha _1^2}{2}\right) t+\alpha _1 W_{1,t}\right\} , \nonumber \\ M_2(t)&= M_2(0)\exp \left\{ \left( -\mu -\frac{\alpha _2^2}{2}\right) t+\alpha _2 W_{2,t}\right\} \end{aligned}$$

and $M_1(0),M_2(0)\ge 0$, the processes $\xi _1(t)$, $\xi _2(t)$ are non-negative.

### Biological Interpretation of the Noise in the Model

There are different ways to introduce stochastic perturbations into a continuous model. In some cases, stochastic perturbations might be computed as a limit of the discrete random process. In population dynamics, a random birth–death process is usually the base of such consideration.

On the other hand, a non-degenerated diffusion in a system of stochastic differential equations, which is proportional to the variables, may be understood as an environmental stochastic perturbation. Similarly to papers (Allen 2003; Arnold et al. 1979; Dalal et al. 2008) a stochastic noise in the model (8) is understood as an environmental perturbation which act independently and proportionally on each population. Noise in the model (8) may be introduced as a limit of birth–death process but in our model it would be difficult to justify it. It is related to the macroscopic response of the population for environmental perturbation. Additionally, the noise in the first equation of system (8) might be understood as an environmental stochastic noise that has an impact on the death and proliferation process. We also assume that this noise has no impact on the regulation of the differentiation process. Independence of the noise in system (8) is understood as a lack of correlation between Wiener processes $W_{1,t}$, $W_{2,t}$. The case that the processes $W_{1,t}$, $W_{2,t}$ are correlated is much more complicated and needs different mathematical methods to be investigated hence, we omit this case in this paper.

## Analytical Results

The main result presented in this chapter is related to the long-time behaviour of the solution of the model (8). We investigate the evolution of the probability density $u(t,x,y)$ of the stochastic process $(\xi _1(t),\xi _2(t))$. Since we assumed that the diffusion process $(\xi _1(t),\xi _2(t))$ is not degenerate, hence by the remark (13) in Appendix, the transition probability function $P(t,\xi _{1,0},\xi _{2,0},A)$ of the process $(\xi _1(t),\xi _2(t))$ is defined as follows

15 $$\begin{aligned} P(t,\xi _{1,0},\xi _{2,0},A)=\mathrm{{Prob}}\bigg ((\xi _1(t),\xi _2(t))\in A\big | (\xi _1(0),\xi _2(0))=(\xi _{1,0},\xi _{2,0})\bigg ) \end{aligned}$$

and has a smooth density. The evolution of the density of the diffusion process is related to a Markov semigroup $\{P(t)\}_{t\ge 0}$ in the following way

16 $$\begin{aligned} P(t)v(x,y)&= u(t,x,y) \quad \mathrm{ for }\; t>0, \end{aligned}$$

where $v(x,y)$ is an initial probability density of the process $(\xi _1(t),\xi _2(t))$. We say that the Markov semigroup is asymptotically stable if and only if there exists a unique time independent density $u^*(x,y)$ such that

17 $$\begin{aligned} \lim _{t\rightarrow \infty } \Vert P(t)v(x,y)-u^*(x,y)\Vert =0. \end{aligned}$$

It is equivalent to the convergence of the distribution of the process $(\xi _1(t),\xi _2(t))$ to the unique stationary distribution for time tending to infinity.

The following proposition provides the necessary condition for the coefficients of the model (8) to guarantee the asymptotic stability of the related Markov semigroup.

### **Proposition 1**

Let $(\xi _1(t),\xi _2(t))$ be a solution of the system (8) and assume that $(2a-1)p-\frac{\alpha _1^2}{2}>0$. Then, the Markov semigroup $\{P(t)\}_{t\ge 0}$ related to the process $(\xi _1(t),\xi _2(t))$ is asymptotically stable.

The condition $(2a-1)p-\frac{\alpha _1^2}{2}>0$ is essential for the model (8). If it is not satisfied, the process $\xi _1$ in the model (8) tends to zero almost sure. And from the fact that the growth of the process $\xi _2$ depends only of the level of the process $\xi _1$, we may conclude that the process $\xi _2(t)$ tends to zero almost sure. Analytical consideration of this fact and the proof of the proposition 1 are presented in Appendix. The proposition 1 shows an important fact that under the condition $(2a-1)p-\frac{\alpha _1^2}{2}>0$, the distribution of the populations stabilises and tends to the stationary distribution.

## Numerical Simulation

In this chapter, we present some numerical simulations of the solutions of the two versions of model (7). As it is mentioned in the previous chapters, model (7) might be considered with several values of $n$. We present two possibilities, the $2$-dimensional model and the $8$-dimensional version. We choose the $8$-dimensional version because of its direct connection to the process of differentiation of white blood cells. Its deterministic realisation and application to hematopoietic reconstitution are presented in Marciniak-Czochra and Stiehl (2012).

The simulations are done using the Matlab R2007b, and SDE Toolbox created by Picchini (2007). The stochastic trajectories were approximated using Euler–Maruyama scheme (Picchini 2007) while the approximation of mean values is done using Monte–Carlo simulations.

All the simulations present mean trajectory and quartiles of $150$ realisations of solutions of the model (7) and histograms created on a base of realisation of $150$ trajectories at the time $T_{\mathrm{{end}}}$. The values of parameters of the model (7) are taken from Marciniak-Czochra and Stiehl (2012), where three different sets of values are proposed. Presented simulations are done for one of those sets of parameters while in the other cases the general behaviour is similar.

**Fig. 1:**
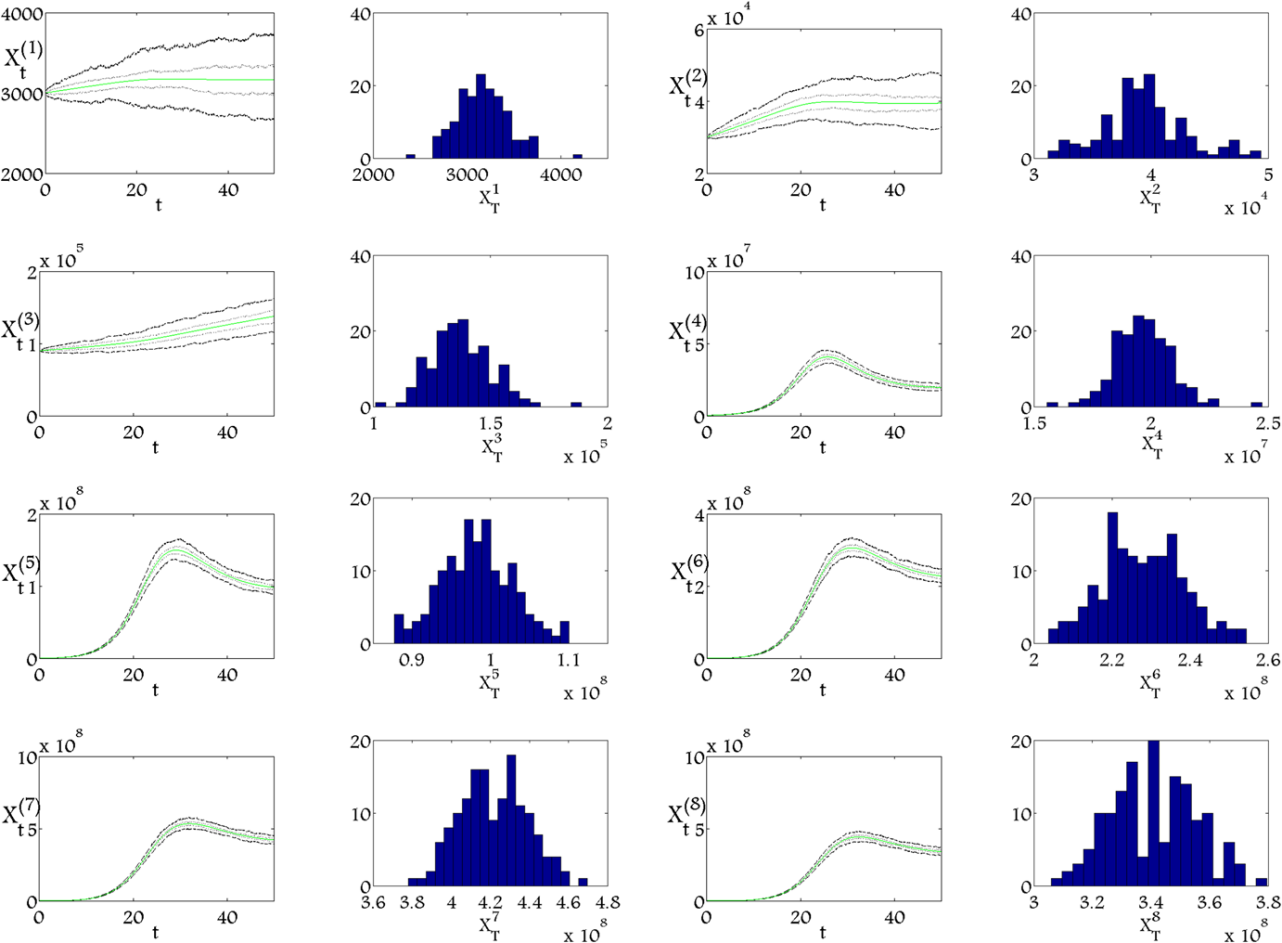
Mean value, $q_1$, $q_3$ quartiles and histograms of 150 trajectories of stochastic processes in time $T_\mathrm{{end}}=50$ of $8$-dimensional version of the model (7) with small diffusion coefficients, where $a_1=0.735$, $a_2=0.7298$, $a_3=0.7245$, $a_4=0.7140$, $a_5=0.5775$, $a_6=0.4725$, $a_7=0.3675$, $p_1=0.006$, $p_2=0.03$, $p_3=0.18$, $p_4=0.6$, $p_5=0.65$, $p_6=1$, $p_7=1.5$, $\mu _1=\mu _2=\mu _3=\mu _4=\mu _5=\mu _6=\mu _7=0$, $\mu _8=2.77$, $\alpha _1=0.011$, $\alpha _2=0.013$, $\alpha _3=0.015$, $\alpha _4=0.017$, $\alpha _5=0.019$, $\alpha _6=0.021$, $\alpha _7=0.023$, $\alpha _8=0.025$, $k=1.28 \times 10^{-9}$

We present three numerical simulations of the stochastic model with $n=8$ for three different sets of diffusion coefficients. Figure 1 depicts the simulation with small diffusion coefficients for all population. It is well known that the realisation of the process with small diffusion coefficients is similar to the solution of deterministic model (Freŏdlin and Wentzell 1998).

**Fig. 2:**
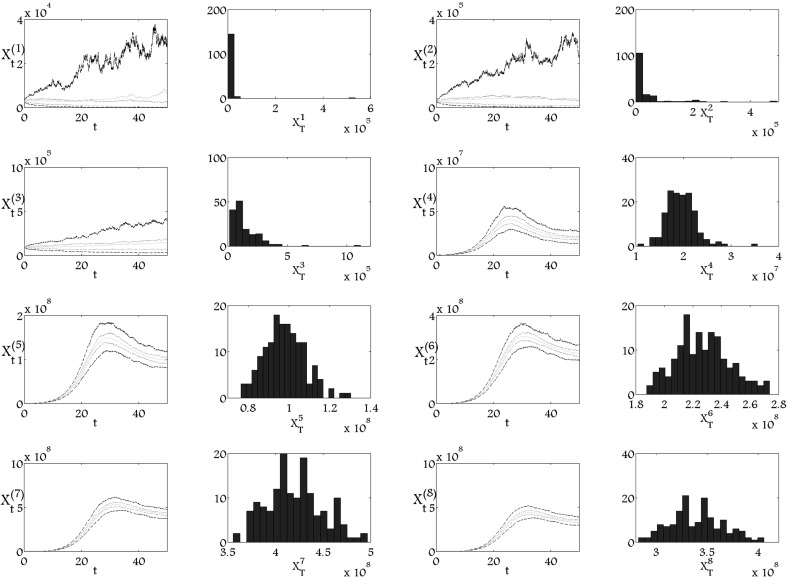
Mean value, $q_1$, $q_3$ quartiles and histograms of 150 trajectories of stochastic processes in time $T_\mathrm{{end}}=50$ of $8$-dimensional version of the model (7) with large diffusion coefficients for subpopulations $\xi ^1_t$, $\xi ^2_t$, $\xi ^3_t$, where $a_1=0.735$, $a_2=0.7298$, $a_3=0.7245$, $a_4=0.7140$, $a_5=0.5775$, $a_6=0.4725$, $a_7=0.3675$, $p_1=0.006$, $p_2=0.03$, $p_3=0.18$, $p_4=0.6$, $p_5=0.65$, $p_6=1$, $p_7=1.5$, $\mu _1=\mu _2=\mu _3=\mu _4=\mu _5=\mu _6=\mu _7=0$, $\mu _8=2.77$, $\alpha _1=0.185$, $\alpha _2=0.18$, $\alpha _3=0.1$, $\alpha _4=0.05$, $\alpha _5=0.03$, $\alpha _6=0.025$, $\alpha _7=0.02$, $\alpha _8=0.015$, $k=1.28\times 10^{-9}$

**Fig. 3:**
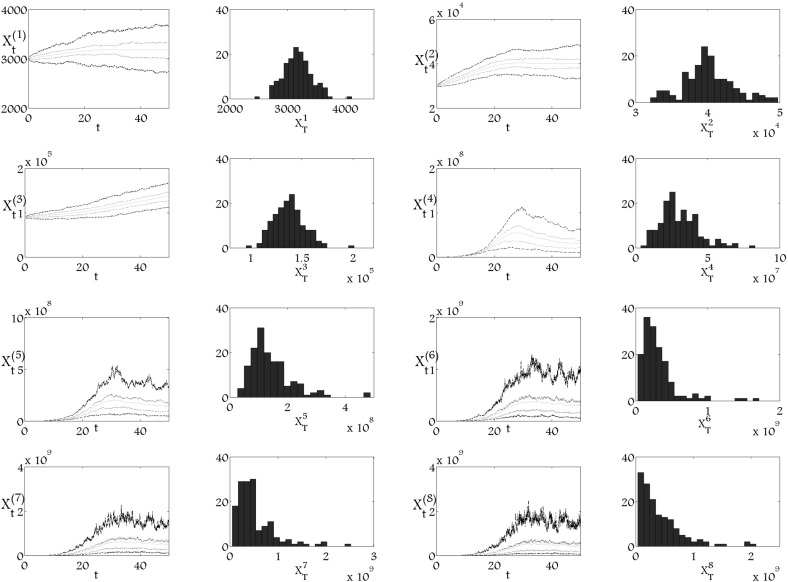
Mean value, $q_1$, $q_3$ quartiles and histograms of 150 trajectories of stochastic processes in time $T_\mathrm{{end}}=50$ of $8$-dimensional version of the model (7) with large diffusion coefficients for subpopulations $\xi ^5_t$, $\xi ^6_t$, $\xi ^7_t$, $\xi ^8_t$, where $a_1=0.735$, $a_2=0.7298$, $a_3=0.7245$, $a_4=0.7140$, $a_5=0.5775$, $a_6=0.4725$, $a_7=0.3675$, $p_1=0.006$, $p_2=0.03$, $p_3=0.18$, $p_4=0.6$, $p_5=0.65$, $p_6=1$, $p_7=1.5$, $\mu _1=\mu _2=\mu _3=\mu _4=\mu _5=\mu _6=\mu _7=0$, $\mu _8=2.77$, $\alpha _1=0.01$, $\alpha _2=0.012$, $\alpha _3=0.018$, $\alpha _4=0.03$, $\alpha _5=0.18$, $\alpha _6=0.5$, $\alpha _7=0.6$, $\alpha _8=1$, $k=1.28\times 10^{-9}$

In simulations presented in Fig. 2, we increase the diffusion coefficients for the stem cells population and populations at first two stages of maturation. A large noise of first three populations leads to the extinction. In this description by ‘extinction’ we mean a convergence of the expected value to zero. However, the interesting observation is that the noise added to the first stages of differentiation does not have any influence on the population of mature cells and the populations at late stages of differentiation. On the other hand, Fig. 3 presents the simulations where we increased the noise for the population of mature cells and the three last stages of differentiation. One may notice that the effect seen in Fig. 2 is obtained in Fig. 3. While the addition of noise disturbs the processes at last stages of differentiation, the first stages of differentiation seem to be insensitive for such perturbation. We might form a conclusion of those three simulations that the effect of noise directed to some subpopulation is dumped at the sufficiently distant subpopulation.

**Fig. 4:**
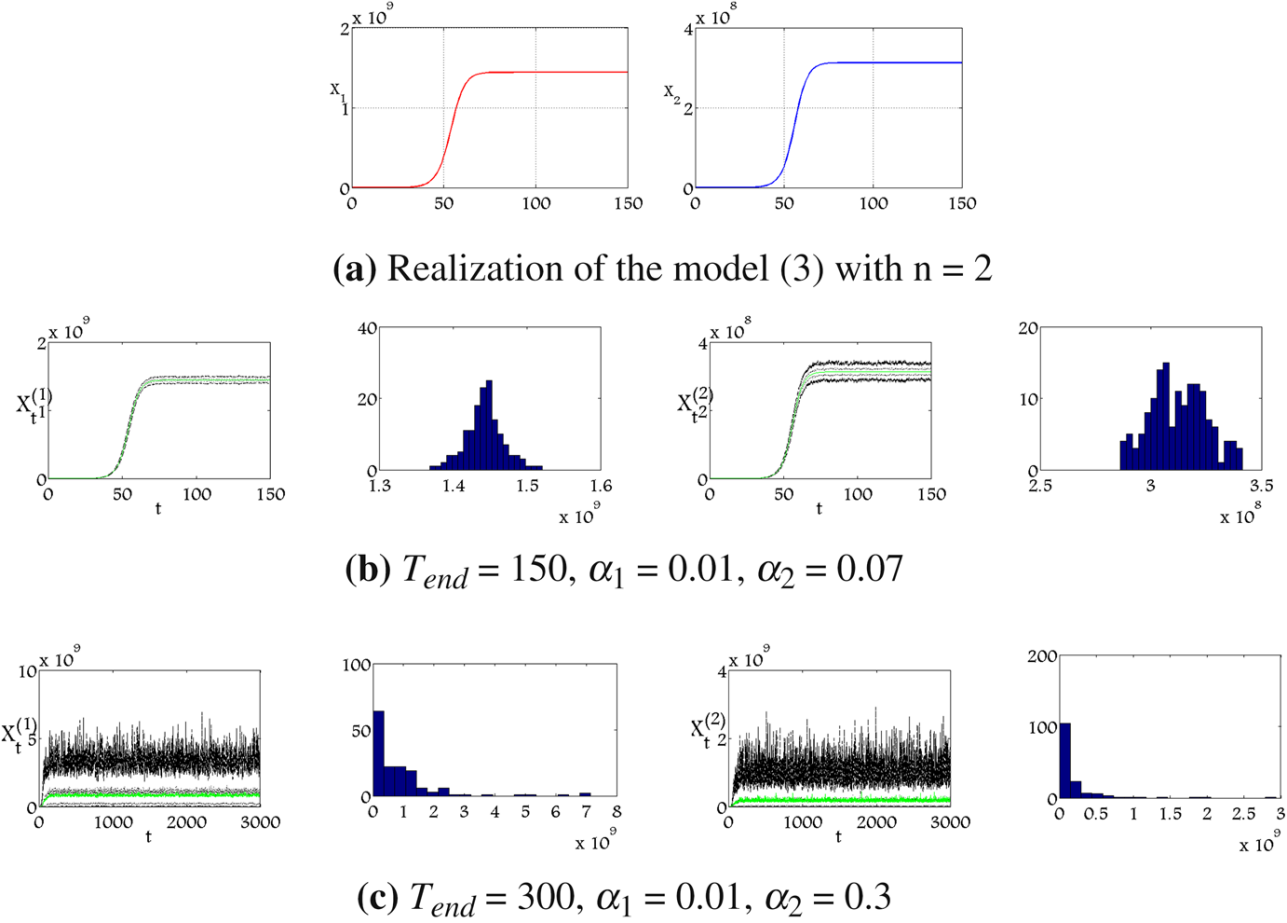
Mean value, $q_1$, $q_3$ quartiles and histograms of 150 trajectories of stochastic processes in time $T_\mathrm{{end}}$ of $2$-dimensional version of the model (8), where $a=0.7$, $p=0.6$, $\mu =2.77$, $k=1.28\times 10^{-9}$

Figure 4 presents a realisation of the $2$-dimensional deterministic and stochastic version of the model (7). It is seen that in the Fig. 4b we present the case where perturbations applied to the subpopulations of undifferentiated cells and mature cells are relatively small consequently, the mean value of 150 trajectories of the stochastic process is almost indifferent to the numerical solution of the equivalent deterministic model shown in the Fig. 4a. In the Fig.  4c one can notice that the level of the noise that is required to lead most paths to zero is relatively large and even in this figure there are still many of trajectories that reach a high level. One can observe that even in this case the mean value of the simulated trajectories stabilises at some time $t$, which is a consequence of the asymptotic stability of the related Markov process.

### Conclusions

Simulations presented in Figs. 1, 2 and 3 show the behaviour of the stochastic model (7) and these need to be interpreted together with the form of the deterministic model. In Fig. 2, we see the extinction of the first three subpopulations. Provided that $a_4>\frac{1}{2}$, the fourth subpopulation should take the role of the stem cells and maintain the whole process. On the other hand, since the third quartiles of the first three subpopulations reach a high level in comparison to the unperturbed case, some of the trajectories also reach large values. However, such a case is not observed in subsequent subpopulations. In Fig. 3 we may observe similar effects. The four last subpopulations are perturbed, although, mean values and quartiles of the trajectories of the first three subpopulations are similar to those presented in Fig. 1. Since some of the realisations of the last subpopulation reach very low levels, the negative feedback should lead to an increase of the stem cell population; however, this is not observed in the stochastic simulation. In Fig. 3, we note that some of the trajectories of the fourth subpopulation reach twice level observed in Fig.  1. Therefore, in the case when the last subpopulatons are perturbed, subpopulations which are the nearest to the maturation level are mobilized to stabilize the process.

## Figures and Tables

**Fig. 5:**
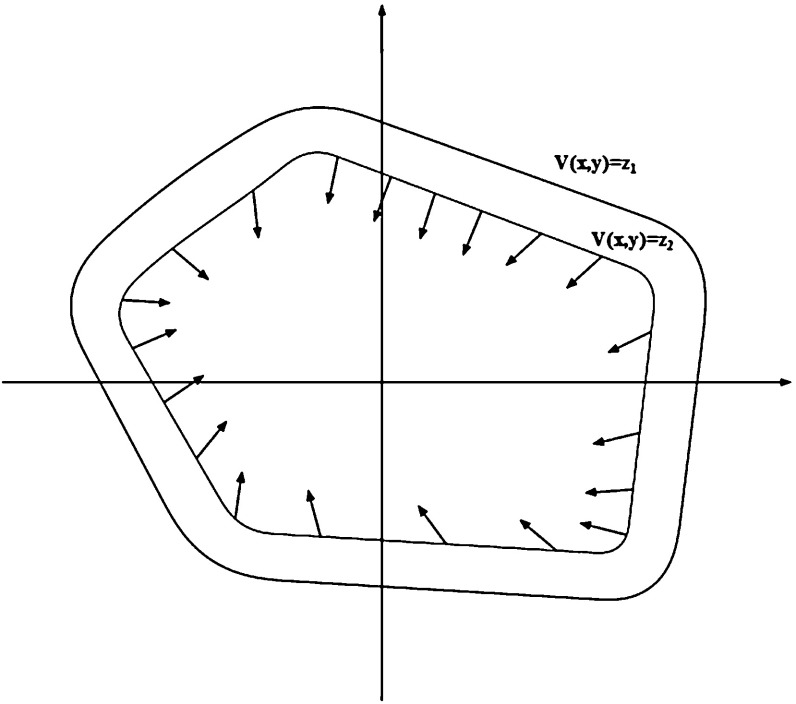
Level lines of the Khasminskií function and the vector field $(g_1(x,y),g_2(x,y))$

## Appendix

### Basic Definitions

Let $(S,\Sigma ,m)$ be a $\sigma$-finite measure space and let $D\subset L^1=L^1(S,\Sigma ,m)$ be the set of densities i.e.

18 $$\begin{aligned} D=\{f\in L^1; f\ge 0, \Vert f\Vert =1\} \end{aligned}$$

We call a linear mapping $P: L^1 \rightarrow L^1$ a Markov operator if $P(D)\subset D$. A family $\{P(t)\}_{t\ge 0}$ of Markov operators is called a Markov semigroup if and only if

1. $$P(0)=id$$
2. $$P(t+s)=P(t)P(s)$$ for every $$t,s\ge 0$$
3. for each $f\in L^1$ the function $t\rightarrow P(t)f$ is continuous with the respect to the $L^1$ norm.

#### **Definition 2**

((Rudnicki 2003, p. 96)) A Markov operator $P$ is called integral if there exists a measurable function $k:S\times S\rightarrow [0,\infty ]$ such that

19 $$\begin{aligned} \int \limits _{S} k(x,y)m(\mathrm{{d}}x)=1 \end{aligned}$$

for almost all $y\in S$ and

20 $$\begin{aligned} Pf(x)= \int \limits _S k(x,y)f(y)m(\mathrm{{d}}y) \end{aligned}$$

for every $f\in D$. We call function $k$ a kernel of the operator $P$.

#### **Definition 3**

((Rudnicki 2003, p. 97)) A Markov semigroup $\{P(t)\}_{t\ge 0}$ is called integral if for every $t>0$ a Markov operator $P(t)$ is an integral Markov operator. That is there exists a measurable function $k:(0,\infty )\times S\times S\rightarrow [0, \infty )$ such that

21 $$\begin{aligned} P(t)f(x)= \int \limits _S k(t,x,y)f(y)m(\mathrm{{d}}y), \quad \mathrm{for }\; \mathrm{every } \quad f\in D. \end{aligned}$$

#### **Definition 4**

A density $f^*$ is called invariant if $P(t)f^*=f^*$ for each $t>0$.

#### **Definition 5**

The Markov semigroup $\{P(t)\}_{t\ge 0}$ is called asymptotically stable if there is an invariant density $f^*$ such that

22 $$\begin{aligned} \lim _{t\rightarrow \infty } \Vert P(t)f-f^*\Vert =0 \quad \mathrm{for\ every }\quad f\in D. \end{aligned}$$

#### *Remark 6*

Let $\{P(t)\}_{t\ge 0}$ be an integral Markov semigroup generated by the equation

23 $$\begin{aligned} \frac{\partial u}{\partial t} =Au \end{aligned}$$

where $A$ is some differential operator. If there exists a unique stationary solution $u^*\in D$ of (23), $u^*>0$, then $\{P(t)\}_{t\ge 0}$ is asymptotically stable which is equivalent to

24 $$\begin{aligned} \lim _{t\rightarrow \infty } \Vert u(t)-u^*\Vert =0. \end{aligned}$$

#### **Definition 7**

A Markov semigroup $\{P(t)\}_{t\ge 0}$ is called sweeping with respect to a set $A\in \Sigma$ if for every $f\in D$

25 $$\begin{aligned} \lim _{t\rightarrow \infty } \int \limits _{A} P(t)f(x)m(\mathrm{{d}}x)=0. \end{aligned}$$

#### **Definition 8**

(*Foguel alternative*) ((Rudnicki et al. 2002, p. 10)) We say that a Markov semigroup $\{P(t)\}_{t\ge 0}$ satisfies the Foguel alternative if it is asymptotically stable or sweeping from a sufficiently large family of sets. For example this family can consist of all compact sets.

#### **Theorem 9**

((Rudnicki 2003, p. 98)) Let $\{P(t)\}_{t\ge 0}$ be an integral Markov semigroup with a continuous kernel $k(t,x,y)$ for every $t>0$. Assume that for every $f\in D$ we have

26 $$\begin{aligned} \int \limits _0^{\infty } P(t)f\mathrm{{d}}t>0. \end{aligned}$$

Then the semigroup $\{P(t)\}_{t\ge 0}$ is asymptotically stable or sweeping with the respect to the family of compact sets.

#### **Definition 10**

((Rudnicki et al. 2002, p. 10)) Consider a Markov semigroup $\{P(t)\}_{t\ge 0}$ and let $A$ be the infinitesimal operator of $\{P(t)\}_{t\ge 0}$. Let $R=(1,A)=(1-A)^{-1}$ be the resolvent operator at point $1$ for an infinitesimal generator of the Markov semigroup $\{P(t)\}_{t\ge 0}$. A measurable function $V:S\rightarrow [0,\infty )$ is called a Khasminskií function for the Markov semigroup $\{P(t)\}_{t\ge 0}$ and a set $Z\in \Sigma$ if there exist $M>0$ and $\varepsilon >0$ such that

27 $$\begin{aligned} \int \limits _S V(x)Rf(x)\mathrm{{d}}m(x)\le \int \limits _S (V(x)-\varepsilon )f(x)\mathrm{{d}}m(x)+\int \limits _S MRf(x)\mathrm{{d}}m(x). \end{aligned}$$

#### **Theorem 11**

((Rudnicki et al. 2002, p. 10)) Let $\{P(t)\}_{t\ge 0}$ be a Markov semigroup generated by the equation

28 $$\begin{aligned} \frac{\partial u}{\partial t}=Au. \end{aligned}$$

Assume that there exist a Khasminskií function for the operator $\{P(t)\}_{t\ge 0}$ and the set $Z$. Then the semigroup $\{P(t)\}_{t\ge 0}$ is asymptotically stable.

Assume that the semigroup $\{P(t)\}_{t\ge 0}$ is generated by the following Fokker–Planck equation

29 $$\begin{aligned} \frac{\partial u}{\partial t} =\sum _{i,j=1}^d\left( \frac{\partial ^2 a_{ij}(x)u}{\partial x_i\partial x_j}\right) +\sum _{i=1}^d\left( \frac{\partial ^2 b_i(x)u}{\partial x_i^2} \right) . \end{aligned}$$

where matrix $a=[a_{ij}]$ is symmetric and positive definite. It means that

30 $$\begin{aligned} \sum _{ij=1}^d a_{ij}(x)\lambda _i\lambda _j\ge 0 \quad {\hbox { for every }} \quad \lambda \ne 0. \end{aligned}$$

For the following theorem let $A$ be a differential operator such as on the right-hand side of the Eq. (29).

#### **Theorem 12**

((Pichór and Rudnicki 1997, p. 59)) Let $\{P(t)\}_{t\ge 0}$ be the Markov semigroup and let $A^*$ be a adjoint operator of the infinitesimal generator $A$ of this semigroup. Assume that there exist a $C^2$ function $V:S\rightarrow [0,\infty )$, $r>0$ and $\varepsilon >0$ such that

31 $$\begin{aligned} \sup _{x\notin B(r)}A^*V(x)\le -\varepsilon . \end{aligned}$$

Then$\{P(t)\}_{t\ge 0}$ is asymptotically stable and $V$ is a Khasminskií function for the Markov semigroup $\{P(t)\}_{t\ge 0}$.

### Approximation Theorem for One Dimensional Itô stochastic Differential Equation

Let $\mu$, $\sigma$ be Lipschitz functions and assume that $\sigma (x)>0$. Let $X(t)$ be the solution of the following SDE with $X(0)=x_0$

32 $$\begin{aligned} \mathrm{{d}}X(t)=\mu (X(t))\mathrm{{d}}t +\sigma (X(t))\mathrm{{d}}W_t. \end{aligned}$$

For every $x$ let

$$\begin{aligned} I_1(x)&= \int \limits _{-\infty }^{x} \exp \left( -\int \limits _{0}^s \frac{2\mu (r)}{\sigma ^2(r)}\mathrm{{d}}r\right) \mathrm{{d}}s, \\ I_2(x)&= \int \limits ^{\infty }_{x} \exp \left( -\int \limits _{0}^s \frac{2\mu (r)}{\sigma ^2(r)}\mathrm{{d}}r\right) \mathrm{{d}}s. \end{aligned}$$

If $I_1(x_0)=\infty$ and $I_2(x_0)=\infty$, then

33 $$\begin{aligned} P(\limsup _{t\rightarrow \infty }X_t=\infty )=P(\limsup _{t\rightarrow \infty }X_t=-\infty )=1. \end{aligned}$$

If $I_1(x_0)<\infty$ and $I_1(x_0)<\infty$, then

34 $$\begin{aligned} P(\limsup _{t\rightarrow \infty }X_t=\infty )&= \frac{I_1(x_0)}{I_1(x_0)+I_2(x_0)}, \nonumber \\ P(\liminf _{t\rightarrow \infty }X_t=-\infty )&= \frac{I_2(x_0)}{I_1(x_0)+I_2(x_0)}. \end{aligned}$$

If $I_1(x_0)<\infty$ and $I_2(x_0)=\infty$, then

35 $$\begin{aligned} P(\lim _{t\rightarrow \infty }X_t=-\infty )=1. \end{aligned}$$

If $I_1(x_0)=\infty$ and $I_2(x_0)<\infty$, then

36 $$\begin{aligned} P(\lim _{t\rightarrow \infty }X_t=\infty )=1. \end{aligned}$$

An invariant density $f_*$ of the semigroup $\{P(t)\}_{t\ge 0}$ related to the Eq. (32) should satisfy the following equation

37 $$\begin{aligned} -\frac{\mathrm{{d}}}{\mathrm{{d}}x}(\mu (x)f_*(x))+\frac{1}{2}\frac{\mathrm{{d}}^2}{\mathrm{{d}}x^2}(\sigma ^2(x)f_*(x))=0 \end{aligned}$$

and invariant density $f_*$ exist if and only if

38 $$\begin{aligned} \int \limits _{\infty }^{\infty }\frac{1}{\sigma ^2(x)}\exp \left( 2\int \limits _0^x \frac{\mu (r)}{\sigma ^2(r)}\mathrm{{d}}r\right) \mathrm{{d}}x<\infty . \end{aligned}$$

Additionally, we may obtain that

39 $$\begin{aligned} f_*(x)=C\frac{1}{\sigma ^2(x)}\exp \left( 2\int \limits _0^x \frac{\mu (r)}{\sigma ^2(r)}\mathrm{{d}}r\right) \end{aligned}$$

for $C>0$. We consider the first equation from the system of stochastic differential Eq. (12)

40 $$\begin{aligned} \mathrm{{d}}N(t)=(2a-1)pN(t)\mathrm{{d}}t +\alpha _1 N(t)\mathrm{{d}}W_t, \end{aligned}$$

where $a>\frac{1}{2}$, $p,\alpha >0$ and $N(0)=n_0$. An invariant density for the diffusion $N(t)$ does not exist what is seen by

41 $$\begin{aligned} I&= \int \limits _{0}^{\infty }\frac{1}{\alpha _1^2} \exp \left( \int \limits _{1}^s \frac{2(2a-1)pr}{\alpha _1^2r^2}\mathrm{{d}}r \right) \mathrm{{d}}s\nonumber \\&= \int \limits _{0}^{\infty }\frac{1}{\alpha _1^2} \exp \left( \frac{2(2a-1)p}{\alpha _1^2}\ln (s) \right) \mathrm{{d}}s\nonumber \\&= \int \limits _{0}^{\infty }\frac{1}{\alpha _1^2}s^{\frac{2(2a-1)p}{\alpha _1^2}}\mathrm{{d}}s=\infty . \end{aligned}$$

To investigate the behaviour of trajectories, we need to notice that $N(t)>0$ a.s. for $n_0 >0$. Hence, we may write the following integrals

42 $$\begin{aligned} I_1(n_0)&= \int \limits _{0}^{n_0} \exp \left( \int \limits _{0}^s \frac{2(2a-1)pr}{-\alpha ^2r^2}\mathrm{{d}}r\right) \mathrm{{d}}s \nonumber \\&= \int \limits _0^{n_0} s^{ \frac{2(2a-1)p}{-\alpha ^2}}\mathrm{{d}}s \nonumber \\ I_2(n_0)&= \int \limits ^{\infty }_{n_0} \exp \left( \int \limits _{0}^s \frac{2(2a-1)pr}{-\alpha ^2r^2}\mathrm{{d}}r\right) \mathrm{{d}}s \nonumber \\&= \int \limits ^{\infty }_{n_0} s^{ \frac{2(2a-1)p}{-\alpha ^2}}\mathrm{{d}}s. \end{aligned}$$

One may notice that for $(2a-1)p-\frac{\alpha ^2}{2}<0$ we get that $I_1(n_0)<\infty$ and $I_2(n_0)=\infty$. Therefore, from (35), we obtain that

43 $$\begin{aligned} P(\lim _{t\rightarrow \infty }N(t)=0)=1 \end{aligned}$$

and for $(2a-1)p-\frac{\alpha ^2}{2}>0$ we obtain that $I_2(x)<\infty$ and $I_1(n_0)=\infty$ so from (36)

44 $$\begin{aligned} P(\lim _{t\rightarrow \infty }N(t)=\infty )=1. \end{aligned}$$

As is shown in Sect. 3 the stochastic process $\xi _1(t)$ is bounded from above by the process $N(t)$ a.s. Therefore, for $(2a-1)p-\frac{\alpha ^2}{2}<0$ we obtain that

45 $$\begin{aligned} P(\lim _{t\rightarrow \infty }\xi _1(t)=0)\ge P(\lim _{t\rightarrow \infty }N(t)=0)=1. \end{aligned}$$

### Proof of the Proposition 1

To show that the statement of the Proposition 1 is true, firstly, we show that the density of the solution of the system (9) is related to an integral Markov semigroup $\{P(t)\}_{t\ge 0}$. From the theorem 9 we know that the integral semigroup satisfies Foguel alternative. Therefore, semigroup $\{P(t)\}_{t\ge 0}$ is asymptotically stable or sweeping from the family of all compact sets. In order to exclude sweeping we construct a function $V$ such that the condition (31) from the proposition 1 is satisfied.

Let $(S,\Sigma , m)=(\mathbb {R}^2,\mathbb {B}(\mathbb {R}^2),m)$, where $m$ is a Lebesgue measure on $\mathbb {R}^2$ and $\mathbb {B}(\mathbb {R}^2)$ is a $\sigma$-field of Borel sets in $\mathbb {R}^2$. We consider the system of differential equations (8), where $c_1(t)$, $c_2(t)$ are stochastic processes adapted to the natural filtration. The solution of this system is non-negative providing the initial processes are non-negative. We may make the following technical substitution $\xi _1=e^x$, $\xi _2=e^y$ and consider the following system of differential equations

46 $$\begin{aligned} \mathrm{{d}}x&= \left( \left( \frac{2a}{1+ke^{y}}-1\right) p -\frac{1}{2}\alpha _1^2\right) \mathrm{{d}}t+\alpha _1\mathrm{{d}}W_{1,t}, \nonumber \\ \mathrm{{d}}y&= \left( 2\left( 1-\frac{a}{1+ke^{y}}\right) pe^{x-y}-\mu -\frac{1}{2}\alpha _2^2\right) \mathrm{{d}}t+\alpha _2\mathrm{{d}}W_{2,t}. \end{aligned}$$

where the process $(x(t),y(t))\in \mathbb {R}^2$. The probability density of the transition probability function of the process $(x(t),y(t))$ is described by the following Fokker–Planck equation

47 $$\begin{aligned} \frac{\mathrm{{d}}\nu }{\mathrm{{d}}t} =\frac{1}{2}\alpha _1^2\frac{\partial ^2\nu }{\partial x^2} + \frac{1}{2}\alpha _2^2\frac{\partial ^2\nu }{\partial y^2} -\frac{\partial (\nu g_1)}{\partial x}-\frac{\partial (\nu g_2)}{\partial y}, \end{aligned}$$

where $g_1$, $g_2:\mathbb {R}^2\rightarrow \mathbb {R}$ are defined as follows

48 $$\begin{aligned} g_1(x,y)&= \left( \frac{2a}{1+ke^{y}}-1\right) p -\frac{1}{2}\alpha _1^2, \nonumber \\ g_2(x,y)&= 2\left( 1-\frac{a}{1+ke^{y}}\right) pe^{x-y}-\mu -\frac{1}{2}\alpha _2^2. \end{aligned}$$

Let $A$ be the differential operator defined as

49 $$\begin{aligned} A\nu =\frac{1}{2}\alpha _1^2\frac{\partial ^2\nu }{\partial x^2} + \frac{1}{2}\alpha _2^2\frac{\partial ^2\nu }{\partial y^2} -\frac{\partial (\nu g_1)}{\partial x}-\frac{\partial (\nu g_2)}{\partial y}. \end{aligned}$$

Then, the adjoint operator of operator $A$ is given by

50 $$\begin{aligned} A^*\nu =\frac{1}{2}\alpha _1^2\frac{\partial ^2\nu }{\partial x^2} + \frac{1}{2}\alpha _2^2\frac{\partial ^2\nu }{\partial y^2} +g_1\frac{\partial \nu }{\partial x}+g_2\frac{\partial \nu }{\partial y}, \end{aligned}$$

Let $\{P(t)\}_{t\ge 0}$ be the Markov semigroup related to the Eq. (47) in the following way

51 $$\begin{aligned} P(t)v_0(x,y)&= \nu (t,x,y) \quad \mathrm{ for }\; t>0 \end{aligned}$$

where $v_0(x,y)$ is the initial probability density of the process $(x(t),y(t))$. Additionally, the operator $A$ defined in (49) is an infinitesimal generator of the semigroup $\{P(t)\}_{t\ge 0}$.

The following remark shows that the semigroup $\{P(t)\}_{t\ge 0}$ generated by the model (8) is integral.

#### *Remark 13*

If the Wiener processes $W_{1,t}$, $W_{2,t}$ are not correlated, then the operator $A$ is elliptic and for every $(x_0,y_0)$ the transition probability function $P(t,x_0,y_0,B)$ of model (46) is absolutely continuous with the respect to the Lebesgue measure and has a density $\nu (t,x_0,y_0,x,y)\in C^2\big ( (0,\infty )\times \mathbb {R}^2\times \mathbb {R}^2\big )$. In addition,

52 $$\begin{aligned} P(t)f(x,y)=\int \limits _{\mathbb {R}^2} \nu (t,x_0,y_0,x,y)f(x,y)\mathrm{{d}}x\mathrm{{d}}y \quad {\hbox { for every }} f\in D. \end{aligned}$$

Hence the semigroup $\{P(t)\}_{t\ge 0}$ is integral.

Therefore, by Theorem 9 the Markov semigroup $\{P(t)\}_{t\ge 0}$ satisfies the Foguel alternative.

According to the Theorem 12, in order to exclude sweeping we need to construct the Khasminskií function for the semigroup $\{P(t)\}_{t\ge 0}$. To simplify the form of the proof, we firstly show the general way of finding Khasminskií function.

Level lines of a Khasminskií function $V(x,y)$ for sufficiently large $R$ and $x^2+y^2>R$ should consist of line segments and circle segments. A construction should be done in the following way. The vector field $(g_1(x,y),g_2(x,y))$ at the level line $V(x,y)=z$ is directed inside the domain $\Gamma =\{(x,y) \mathrm : V(x,y)\le z\}$, see Fig. 5 for graphical interpretation. Since the vector field is directed inside the domain $\Gamma$ we may notice that at the line segments of the Khasminskií function, for some $b_0>0$ the following condition is true

53 $$\begin{aligned} A^*_1V(x,y)=g_1(x,y)\frac{\partial V}{\partial x}+g_2(x,y)\frac{\partial v}{\partial y} \le - b_0. \end{aligned}$$

At the circle segments we may easily check that

54 $$\begin{aligned} 0<\frac{\partial ^2 z}{\partial x^2},\frac{\partial ^2 z}{\partial y^2}<\frac{6}{r}. \end{aligned}$$

Therefore, because $\alpha _1, \alpha _2$ are constant, then for sufficiently large $r$ we may obtain that

55 $$\begin{aligned} A_2^*\nu =\frac{1}{2}\alpha _1^2\frac{\partial ^2\nu }{\partial x^2} + \frac{1}{2}\alpha _2^2\frac{\partial ^2\nu }{\partial y^2}\le \frac{b_0}{2}. \end{aligned}$$

Hence, from (54), (55) we conclude that there exists such $b_0>\varepsilon >0$ that the following condition holds

56 $$\begin{aligned} A^*V(x,y)\le -\varepsilon \end{aligned}$$

for some $r>0$ and $(x,y)\notin B(r)$. Since $\varepsilon >0$ the condtion from the Theorem 12 holds. To finish the proof of asymptotic stability of the Markov semigroup $\{P(t)\}_{t\ge 0}$ we need function $V$ to be $C^2$-function. The following technique presents how to obtain such condition.

We convolute our function $V$ with a function $f^\eta$ constructed in the following way. Let $f^\eta$ be a sufficiently smooth function with compact support contained in a closed ball $B(\eta )\subset \mathbb {R}^2$ with centre in $(0,0)$ and radius $\eta$. Let $K^\eta$ be $\eta$-neighbourhood of the set $K$, $K_1$, respectively. It means that $K^\eta =\{x\in \mathbb {R}^2\mathrm : \inf _{u\in K}\Vert x-u\Vert \le \eta \}$. Hence, let $V^\eta =V*f^\eta$ and then for $x\notin K^\eta$ we obtain

57 $$\begin{aligned} A^*V^\eta&= (A^*(V*f^\eta )(x) = \int \limits _{\mathbb {R}^2} A^*V(x-z)f^\eta (z)dz\nonumber \\&= \int \limits _{B(\eta )}A^*V(x-z)f^\eta (z)dz \le -\varepsilon , \end{aligned}$$

because $x-z \notin K$ for $z\in B(\eta )$. In this way we obtain $C^2$-function $V$ such that the condition from theorem 12 holds and the proof of the preposition 1 is finished. Therefore, the semigroup $\{P(t)\}_{t\ge 0}$ is asymptotically stable.

Exact construction of Khasminskií function is very technical. We have omitted pure algebraic computation and left only important conditions and inequalities. The proof is divided into two parts. In the first one we assume that

58 $$\begin{aligned} \mu +\frac{1}{2}\alpha _2^2-p-\frac{1}{2}\alpha _1^2>0. \end{aligned}$$

In the second one we assume that there exists $n\in \mathbb {N}$ such that

59 $$\begin{aligned} (n-1)\left( \mu +\frac{1}{2}\alpha _2^2\right) -p-\frac{1}{2}\alpha _1^2>0. \end{aligned}$$

At first we show seweral conditions which holds for model (9). Then we present the exact formula of the function $V(x,y)$ and domains $D_1,\dots ,D_{12}$ for related line and circle segments. Consequently we show the boundaries of the derivatives of the function $V$ which lead to estimation of $A^*V$ on each domain $D_1,\dots ,D_{12}$. Similar computation is done for the second case. Ultimately we obtain function $V$ for which $A^*V<-\varepsilon$ for some $\varepsilon >0$.

By virtue of (4), (58) the following inequalities hold for some sufficiently small $\varepsilon >0$

60 $$\begin{aligned}&\mu +\frac{1}{2}\alpha _2^2-p-\frac{1}{2}\alpha _1^2 -2\varepsilon >0, \nonumber \\&\quad (2a-1)p-\frac{1}{2}\alpha _1^2 >\varepsilon . \end{aligned}$$

We fix $\varepsilon >0$ such that the Eq. (60) hold and define the following constants for $r>0$

61 $$\begin{aligned} \gamma&= \ln \left( \frac{\frac{4ap}{\alpha _1^2+2p}-1}{k}\right) , \nonumber \\ \gamma _1&= \ln \left( \frac{\mu +\frac{1}{2}\alpha _2^2-p-\frac{1}{2}\alpha _1^2 -2\varepsilon }{2p}\right) -r, \nonumber \\ \gamma _2&= \ln \left( \frac{\mu +\frac{1}{2}\alpha _2^2+(2a-1)p-\frac{1}{2}\alpha _1^2 +2\varepsilon }{2(1-a)p}\right) +r. \end{aligned}$$

We may notice that there exists $\delta >0$ and sufficiently big $M>0$ such that the following inequalities hold

62 $$\begin{aligned}&\left( \frac{2a}{1+e^y}-1\right) p-\frac{1}{2}\alpha _1^2 <-2\varepsilon \quad \mathrm{for }\; y>\gamma +\delta -r, \nonumber \\&\left( \frac{2a}{1+e^y}-1\right) p-\frac{1}{2}\alpha _1^2 >2\varepsilon \quad \mathrm{for }\; y<\gamma -\delta +r, \nonumber \\&\left( \frac{2a}{1+e^y}-1\right) p-\frac{1}{2}\alpha _1^2 <-3\varepsilon \quad \mathrm{for }\; y>\gamma +M, \nonumber \\&\left( \frac{2a}{1+e^y}-1\right) p-\frac{1}{2}\alpha _1^2 >3\varepsilon \quad \mathrm{for }\; y<\gamma -M. \end{aligned}$$

We note that for every $y>x-\gamma _1$

63 $$\begin{aligned} 2\left( 1-\frac{a}{1+ke^{y}}\right) pe^{x-y}-\mu -\frac{1}{2}\alpha _2^2<-p-\frac{1}{2}\alpha _1^2-2\varepsilon \end{aligned}$$

and for every $y<x-\gamma _2$

64 $$\begin{aligned} 2\left( 1-\frac{a}{1+ke^{y}}\right) pe^{x-y}-\mu -\frac{1}{2}\alpha _2^2>(2a-1)p-\frac{1}{2}\alpha _1^2+2\varepsilon . \end{aligned}$$

Let $z=V(x,y):\mathbb {R}^2\rightarrow [0,\infty )$ be defined as follows

65 $$\begin{aligned} \begin{array}{lllll} &{}(x-z-\gamma -\gamma _1)^4+(y-z-\gamma )^4 = r^4 &{}\quad \mathrm{ for }\; x,y &{}\in &{}D_1, \\ &{}z=y-\gamma - r &{}\quad \mathrm{ for }\; x,y &{}\in &{}D_2, \\ &{}(y-z-\gamma )^4 + (x-y+z-\gamma _1)^4=r^4 &{}\quad \mathrm{ for }\; x,y &{}\in &{}D_3, \\ &{}z=y-x-r+\gamma _1 &{}\quad \mathrm{ for }\; x,y &{}\in &{}D_4, \\ &{}(x+z+\delta -\gamma -\gamma _1)^4+(y-x-z+\gamma _1)^4=r^4 &{}\quad \mathrm{ for }\; x,y &{}\in &{}D_5, \\ &{}z=-x+\gamma +\gamma _1 -\delta -r &{}\quad \mathrm{ for }\; x,y &{}\in &{}D_6,\\ &{}(x+z+\delta -\gamma -\gamma _1)^4+(y+z+\delta -\gamma -\gamma _1+\gamma _2)^4=r^4 &{}\quad \mathrm{ for }\; x,y &{}\in &{}D_7,\\ &{}z=-y-\delta +\gamma +\gamma _1-\gamma _2-r &{}\quad \mathrm{ for }\; x,y &{}\in &{}D_8, \\ &{}(y+z+\delta -\gamma -\gamma _1+\gamma _2)^4+(x-y-z-\gamma _1+\delta )^4=r^4 &{}\quad \mathrm{ for }\; x,y &{}\in &{}D_9, \\ &{}z=x-y-\gamma _1+\delta -r &{}\quad \mathrm{ for }\; x,y &{}\in &{}D_{10},\\ &{}(x-z-\gamma -\gamma _1)^4+(y-x+z+\gamma _1-\delta )^4=r^4 &{}\quad \mathrm{ for }\; x,y &{}\in &{}D_{11}, \\ &{}z=x-\gamma -\gamma _1 -r &{}\quad \mathrm{ for }\; x,y &{}\in &{}D_{12}, \end{array} \end{aligned}$$

where domains $D_1,\dots ,D_{12}$ are defined as follows for some $z_0>0$

66 $$\begin{aligned} \begin{array}{lll} D_1{:=}\{(x,y)\in \mathbb {R}^2;&{} y>z_0+\gamma + r, x>z_0+\gamma +\gamma _1, x-\gamma _1 -r <y<x-\gamma _1 +r \}, \\ D_2{:=}\{(x,y)\in \mathbb {R}^2;&{} y>z_0+\gamma + r, x>\gamma +\gamma _1 +r, x-\gamma _1 -r <y\}, \\ D_3{:=}\{(x,y)\in \mathbb {R}^2;&{} y>z_0+\gamma , x<\gamma +\gamma _1 +r, \frac{1}{2}x+\frac{\gamma +\gamma _1+r}{2} +z_0 <y\}, \\ D_4{:=}\{(x,y)\in \mathbb {R}^2;&{} y>x+z_0-\gamma _1+r +\delta +r, x<\gamma +\gamma _1-r, \gamma -\delta +r <y\}, \\ D_5{:=}\{(x,y)\in \mathbb {R}^2;&{} y>2(x+z_0-\gamma _1)-\gamma +\delta +r, \gamma -\delta -r <y<\gamma -\delta +r \}, \\ D_6{:=}\{(x,y)\in \mathbb {R}^2;&{} x<z_0+\gamma +\gamma _1-\delta -z_0-r, x-\gamma _2 +r <y<\gamma -\delta -r \},\\ D_7{:=}\{(x,y)\in \mathbb {R}^2;&{} y<-x-2(z_0+\gamma +\gamma _1-\delta )-\gamma _2+ r, \\ &{} x-\gamma _2 -r <y<x-\gamma _2 +r \}, \\ D_8{:=}\{(x,y)\in \mathbb {R}^2;&{} y<-z_0-\delta +\gamma +\gamma _1-\gamma _2-r, x<2(\gamma _1-\delta ) +\gamma -\gamma _2-r,\\ &{} y<x-\gamma _2 -r \}, \\ D_9{:=}\{(x,y)\in \mathbb {R}^2;&{} y<-z_0-\delta +\gamma +\gamma _1-\gamma _2, x>2(\gamma _1-\delta ) +\gamma -\gamma _2-r, \\ &{} y<\frac{1}{2}(x-\delta +\gamma -\gamma _2)-z_0+\gamma _1 \}, \\ D_{10}{:=}\{(x,y)\in \mathbb {R}^2;&{} y>-z_0-\delta +\gamma +\gamma -1-\gamma _2, x>2(\gamma _1-\delta )+\gamma _2+r, \\ &{}y<\gamma +\delta -r \}, \\ D_{11}{:=}\{(x,y)\in \mathbb {R}^2;&{} y>\gamma +\delta -r, x>z_0+\gamma +\gamma _1, y<2(x-z_0-\gamma _1)-\gamma +\delta \}, \\ D_{12}{:=}\{(x,y)\in \mathbb {R}^2;&{} y>\gamma +\delta + r, x>z_0+\gamma +\gamma _1+r, y<x-\gamma _1 -r \}. \end{array} \end{aligned}$$

We can estimate the first and the second order derivative of the function $z=V(x,y)$ in the domains $D_1,\dots ,D_{12}$ and obtain the following inequalities

67 $$\begin{aligned} 0<\frac{\partial z}{\partial x}, \frac{\partial z}{\partial y}<1, 0<\frac{\partial ^2 z}{\partial x^2}, \frac{\partial ^2 z}{\partial y^2}<\frac{6}{r}\quad \mathrm{ for }\; x,y&\in D_1, \nonumber \\ \frac{\partial z}{\partial x}=0, \frac{\partial z}{\partial y}=1 \mathrm{ for }\; x,y&\in D_2, \nonumber \\ -1<\frac{\partial z}{\partial x}<0, \frac{\partial z}{\partial y}=1, 0<\frac{\partial ^2 z}{\partial x^2}<\frac{6}{r}, \frac{\partial ^2 z}{\partial y^2}=0 \quad \mathrm{ for }\; x,y&\in D_3, \nonumber \\ \frac{\partial z}{\partial x}=-1, \frac{\partial z}{\partial y}=1 \quad \mathrm{ for }\; x,y&\in D_4, \nonumber \\ \frac{\partial z}{\partial x}=-1, 0<\frac{\partial z}{\partial y}<1, \frac{\partial ^2 z}{\partial x^2}=0, 0<\frac{\partial ^2 z}{\partial y^2}<\frac{6}{r}\quad \mathrm{ for }\; x,y&\in D_5, \nonumber \\ \frac{\partial z}{\partial x}=-1, \frac{\partial z}{\partial y}=0 \quad \mathrm{ for }\; x,y&\in D_6, \nonumber \\ -1<\frac{\partial z}{\partial x}, \frac{\partial z}{\partial y}<0, 0<\frac{\partial ^2 z}{\partial x^2}, \frac{\partial ^2 z}{\partial y^2}<\frac{6}{r} \quad \mathrm{ for }\; x,y&\in D_7, \nonumber \\ \frac{\partial z}{\partial x}=0, \frac{\partial z}{\partial y}=-1\quad \mathrm{ for }\; x,y&\in D_8, \nonumber \\ 0<\frac{\partial z}{\partial x}<1, \frac{\partial z}{\partial y}=-1, 0<\frac{\partial ^2 z}{\partial x^2}<\frac{6}{r}, \frac{\partial ^2 z}{\partial y^2}=0\quad \mathrm{ for }\; x,y&\in D_9, \nonumber \\ \frac{\partial z}{\partial x}=1, \frac{\partial z}{\partial y}=-1 \quad \mathrm{ for }\; x,y&\in D_{10}, \nonumber \\ \frac{\partial z}{\partial x}=1, -1<\frac{\partial z}{\partial y}=0, \frac{\partial ^2 z}{\partial x^2}=0, 0<\frac{\partial ^2 z}{\partial y^2}<\frac{6}{r} \quad \mathrm{ for }\; x,y&\in D_{11}, \nonumber \\ \frac{\partial z}{\partial x}=1, \frac{\partial z}{\partial y}=0\quad \mathrm{ for }\; x,y&\in D_{12}. \end{aligned}$$

One can also notice that in the domains $D_1,D_7$ the following equalities are obtained

68 $$\begin{aligned} \begin{array}{llll} \frac{\partial z}{\partial x} +\frac{\partial z}{\partial y}=1&{}\quad \mathrm{ for }\; x,y &{}\in &{} D_1, \\ \frac{\partial z}{\partial x} +\frac{\partial z}{\partial y}=-1&{}\quad \mathrm{ for }\; x,y &{}\in &{} D_7. \end{array} \end{aligned}$$

Using (62) we can estimate functions $g_1,g_2$ defined in (48) in the domains $D_1, \dots ,D_{12}$

69 $$\begin{aligned} g_1(x,y)&< -3\varepsilon \quad \mathrm{in }\; D_1, D_2, D_5, D_6, \nonumber \\ g_1(x,y)&< -p -\frac{1}{2}\alpha _1^2 -2\varepsilon \quad \mathrm{in } \;D_3, D_4, \nonumber \\ g_1(x,y)&> 3\varepsilon \quad \mathrm{in }\; D_7, D_8, D_{10}, D_{11}, D_{12}, \nonumber \\ g_1(x,y)&> (2a-1)p-\frac{1}{2}\alpha _1^2+2\varepsilon \quad \mathrm{in }\; D_9, D_{10},\nonumber \\ g_2(x,y)&< -3\varepsilon \quad \mathrm{in }\; D_1, D_2, D_3, \nonumber \\ g_2(x,y)&< -2\varepsilon \quad \mathrm{in }\; D_4, D_{11}, D_{12}, \nonumber \\ g_2(x,y)&> 2\varepsilon \quad \mathrm{in }\; D_5, D_6,\nonumber \\ g_2(x,y)&> 3\varepsilon \quad \mathrm{in }\; D_7, D_8, D_9. \end{aligned}$$

By virtue of properties (67), (68) and (69) and assuming that $A^*$ is an infinitesimal generator of the Markov semigroup $\{P(t)\}_{t\ge 0}$ related to the model (8), $r>2\max \{\frac{1}{\varepsilon }\alpha _1^2,\frac{1}{\varepsilon }\alpha _2^2\}$, $z_0>0$ is sufficiently large, we obtain that

70 $$\begin{aligned} A^*V&< -3\varepsilon \left( \frac{\partial z}{\partial x}+\frac{\partial z}{\partial y}\right) +\frac{3}{r}(\alpha _1^2+\alpha _2^2)<-\varepsilon \quad \mathrm{ for }\; x,y \in D_1, \nonumber \\ A^*V&< -3\varepsilon \frac{\partial z}{\partial y}<-\varepsilon \quad \mathrm{ for }\; x,y \in D_2, \nonumber \\ A^*V&< \frac{\partial z}{\partial x}\left( \left( \frac{2a}{1+ke^y}-1\right) p-\frac{1}{2}\alpha ^2_1\right) -p-\frac{1}{2}\alpha _1^2-2\varepsilon +\frac{3}{r}\alpha _1^2 <-\varepsilon \quad \mathrm{ for }\; x,y \in D_3, \nonumber \\ A^*V&< -\left( \left( \frac{2a}{1+ke^y}-1\right) p-\frac{1}{2}\alpha ^2_1\right) -p-\frac{1}{2}\alpha _1^2-2\varepsilon <-\varepsilon \quad \mathrm{ for }\; x,y \in D_4, \nonumber \\ A^*V&< -\left( \left( \frac{2a}{1+ke^{\gamma -\delta +r}}-1\right) p-\frac{1}{2}\alpha ^2_1\right) +\frac{3}{r}\alpha _2^2 <-\varepsilon \quad \mathrm{ for }\; x,y \in D_5, \nonumber \\ A^*V&< -\left( \left( \frac{2a}{1+ke^{\gamma -\delta +r}}-1\right) p-\frac{1}{2}\alpha ^2_1\right) <-\varepsilon \quad \mathrm{ for }\; x,y \in D_6, \nonumber \\ A^*V&< 3\varepsilon \left( \frac{\partial z}{\partial x}+\frac{\partial z}{\partial y}\right) +\frac{3}{e}(\alpha _1^2+\alpha _2^2) <-\varepsilon \quad \mathrm{ for }\; x,y \in D_7, \nonumber \\ A^*V&< -3\varepsilon \frac{\partial z}{\partial y} <-\varepsilon \quad \mathrm{ for }\; x,y \in D_8, \nonumber \\ A^*V&< \frac{\partial z}{\partial x}\left( \left( \frac{2a}{1+ke^y}-1\right
) p-\frac{1}{2}\alpha ^2_1\right) -(2a-1)p+\frac{1}{2}+\alpha _1^2-2\varepsilon +\frac{3}{r}\alpha _1^2 \nonumber \\&< -\varepsilon \quad \mathrm{ for }\; x,y \in D_9, \nonumber \\ A^*V&< \frac{\partial z}{\partial x}\left( \left( \frac{2a}{1+ke^y}-1\right) p-\frac{1}{2}\alpha ^2_1\right) -(2a-1)p+\frac{1}{2}\alpha _1^2-2\varepsilon <-\varepsilon \quad \mathrm{ for }\; x,y \in D_{10}, \nonumber \\ A^*V&< \left( \left( \frac{2a}{1+ke^{\gamma +\delta -r}}-1\right) p-\frac{1}{2}\alpha ^2_1\right) +\frac{3}{r}\alpha _2^2<-\varepsilon \quad \mathrm{ for }\; x,y \in D_{11}, \nonumber \\ A^*V&< \left( \left( \frac{2a}{1+ke^{\gamma +\delta -r}}-1\right) p-\frac{1}{2}\alpha ^2_1\right) <-\varepsilon \quad \mathrm{ for }\; x,y \in D_{12}. \end{aligned}$$

Inequalities (70) show that $A^*V<-\varepsilon$ for $x,y\notin K$, where $K\subset \mathbb {R}^2$ is a compact set bounded by the following lines

71 $$\begin{aligned} y&= z_0+\gamma -r, \quad y=z_0+x+r-\gamma _1, \nonumber \\ x&= -z_0+\gamma +\gamma _1-\delta -r, \quad y=-z_0-\delta +\gamma +\gamma _1 -\gamma _2-r, \nonumber \\ y&= -z_0+x-\gamma _1+\delta -r, \quad x=z_0+\gamma +\gamma _1 +r. \end{aligned}$$

Under the condition (59) we construct function $z=V(x,y)$ in the following way

72 $$\begin{aligned} (x-z-\gamma -\gamma _1)^4+(y-z-\gamma )^4 = r^4 \quad \mathrm{ for }\; x,y&\in D_1, \nonumber \\ z=y-\gamma + r \quad \mathrm{ for }\; x,y&\in D_2, \nonumber \\ (y-z-\gamma )^4 + (x-ny+(n+1)(z+\gamma ))^4=r^4 \quad \mathrm{ for }\; x,y&\in D_3, \nonumber \\ z=\frac{ay-x-r}{a+1}-\gamma \quad \mathrm{ for }\; x,y&\in D_4, \nonumber \\ (x+(n+1)(z+\gamma )-n(\gamma -\delta ))^4+(ay-x-(n+1(z+\gamma ))^4=r^4 \quad \mathrm{ for }\; x,y&\in D_5, \nonumber \\ z=\frac{(\gamma -\delta )n-x-r}{n+1}-\gamma \quad \mathrm{ for }\; x,y&\in D_6, \nonumber \\ (x+(n+1)(z+\gamma )-n(\gamma -\delta ))^4+(y+(n+1)(z+\gamma )-n(\gamma -\delta )+\gamma _2)^4&= r^4 \nonumber \\ \quad \mathrm{ for }\; x,y&\in D_7, \nonumber \\ z=\frac{-(n+1)(z+\gamma )+n(\gamma -\delta )-\gamma _2-r}{n+1}-\gamma \quad \mathrm{ for }\; x,y&\in D_8, \nonumber \\ (y+(n+1)(z+\gamma )-n(\gamma -\delta )+\gamma _2)^4+(x-y-z-\gamma _1-\delta )^4=r^4 \quad \mathrm{ for }\; x,y&\in D_9, \nonumber \\ z=x-y-\gamma _1-\delta -r \quad \mathrm{ for }\; x,y&\in D_{10}, \nonumber \\ (x-z-\gamma -\gamma _1)^4+(y-x+z+\gamma _1+\delta )^4=r^4 \quad \mathrm{ for }\; x,y&\in D_{11}, \nonumber \\ z=x-\gamma -\gamma _1 -r \quad \mathrm{ for }\; x,y&\in D_{12},\nonumber \\ \end{aligned}$$

where $\gamma _1=\ln (\frac{\mu +\frac{1}{2}\alpha _2^2}{2p})-r$ and $\gamma$, $\gamma _2$, $\delta$ are the same as in (61). The domains $D_1,\dots ,D_{12}$ are defined as follows

73 $$\begin{aligned} D_1{:=}\{(x,y)\in \mathbb {R}^2;&y>z_0+\gamma , x-\gamma _1 -r <y<x-\gamma _1 +r \}, \nonumber \\ D_2{:=}\{(x,y)\in \mathbb {R}^2;&y>z_0+\gamma + r, y>-x+(n+1)r, x-\gamma _1 +r <y\}, \nonumber \\ D_3{:=}\{(x,y)\in \mathbb {R}^2;&y>z_0+\gamma , y<-x+(n+1)r, y>-x-r\}, \nonumber \\ D_4{:=}\{(x,y)\in \mathbb {R}^2;&y<-x-r, \gamma -\delta +\frac{r}{n} <y, y>\frac{x+(n+1)(z_0+\gamma )}{n}+\frac{r}{n}\} \nonumber \\ D_5{:=}\{(x,y)\in \mathbb {R}^2;&\gamma -\delta +\frac{r}{n} >y, \gamma -\delta -\frac{r}{n} <y \},\nonumber \\ D_6{:=}\{(x,y)\in \mathbb {R}^2;&\gamma -\delta -\frac{r}{n} >y, x-\gamma _2 +r <y\},\nonumber \\ D_7{:=}\{(x,y)\in \mathbb {R}^2;&x-\gamma _2 -r <y<x-\gamma _2 +r \}, \nonumber \\ D_8{:=}\{(x,y)\in \mathbb {R}^2;&y<\frac{n+1}{n}x+\frac{r+\gamma -\delta \gamma _2}{n+1}-\gamma _1+\gamma +\delta , y<x-\gamma _2 -r \}, \nonumber \\ D_9{:=}\{(x,y)\in \mathbb {R}^2;&y>\frac{n+1}{n}x+\frac{r+\gamma -\delta \gamma _2}{n+1}-\gamma _1+\gamma +\delta , \nonumber \\&y<\frac{n+1}{n}x+\frac{r+\gamma -\delta \gamma _2}{n+1}-\gamma _1+\gamma +\delta +r \}, \nonumber \\ D_{10}{:=}\{(x,y)\in \mathbb {R}^2;&y>\frac{n+1}{n}x+\frac{r+\gamma -\delta \gamma _2}{n+1}-\gamma _1+\gamma +\delta +r, y<\gamma +\delta -r \}, \nonumber \\ D_{11}{:=}\{(x,y)\in \mathbb {R}^2;&y>\gamma +\delta -r, y<\gamma +\delta +r, x>z_0+\gamma +\gamma _1 \},\nonumber \\ D_{12}{:=}\{(x,y)\in \mathbb {R}^2;&y>\gamma +\delta + r, x>z_0+\gamma +\gamma _1+r, y<x-\gamma _1 -r \}.\nonumber \\ \end{aligned}$$

An approximation of the first and the second order derivative of the function $z=V(x,y)$ in domains $D_1,\dots ,D_{12}$ is following

74 $$\begin{aligned} 0<\frac{\partial z}{\partial x}, \frac{\partial z}{\partial y}<1, 0<\frac{\partial ^2 z}{\partial x^2}, \frac{\partial ^2 z}{\partial y^2}<\frac{6}{r}\quad \mathrm{ for }\; x,y&\in D_1, \nonumber \\ \frac{\partial z}{\partial x}=0, \frac{\partial z}{\partial y}=1 \mathrm{ for }\; x,y&\in D_2, \nonumber \\ -\frac{1}{n+1}<\frac{\partial z}{\partial x}<0, \frac{n}{n+1}<\frac{\partial z}{\partial y}<1, 0<\frac{\partial ^2 z}{\partial x^2}<\frac{6}{r} \quad \mathrm{ for }\; x,y&\in D_3, \nonumber \\ \frac{\partial z}{\partial x}=-\frac{1}{n+1}, \frac{\partial z}{\partial y}=\frac{n}{n+1} \quad \mathrm{ for }\; x,y&\in D_4, \nonumber \\ \frac{\partial z}{\partial x}=-\frac{1}{n+1}, 0<\frac{\partial z}{\partial y}<\frac{n}{n+1}, \frac{\partial ^2 z}{\partial x^2}=0, 0<\frac{\partial ^2 z}{\partial y^2}<\frac{6}{r}\quad \mathrm{ for }\; x,y&\in D_5, \nonumber \\ \frac{\partial z}{\partial x}=-\frac{1}{n+1}, \frac{\partial z}{\partial y}=0 \quad \mathrm{ for }\; x,y&\in D_6, \nonumber \\ -\frac{1}{n+1}<\frac{\partial z}{\partial x}, \frac{\partial z}{\partial y}<0, 0<\frac{\partial ^2 z}{\partial x^2}, \frac{\partial ^2 z}{\partial y^2}<\frac{6}{r(n+1)} \quad \mathrm{ for }\; x,y&\in D_7, \nonumber \\ \frac{\partial z}{\partial x}=0, \frac{\partial z}{\partial y}=-\frac{1}{n+1}\quad \mathrm{ for }\; x,y&\in D_8, \nonumber \\ 0<\frac{\partial z}{\partial x}<1, -1<\frac{\partial z}{\partial y}<-\frac{1}{n+1}, 0<\frac{\partial ^2 z}{\partial x^2}<\frac{6}{r(n+1)}\quad \mathrm{ for }\; x,y&\in D_9, \nonumber \\ \frac{\partial z}{\partial x}=1, \frac{\partial z}{\partial y}=-1 \quad \mathrm{ for }\; x,y&\in D_{10}, \nonumber \\ \frac{\partial z}{\partial x}=1, -1<\frac{\partial z}{\partial y}=0, \frac{\partial ^2 z}{\partial x^2}=0, 0<\frac{\partial ^2 z}{\partial y^2}<\frac{6}{r} \quad \mathrm{ for }\; x,y&\in D_{11}, \nonumber \\ \frac{\partial z}{\partial x}=1, \frac{\partial z}{\partial y}=0\quad \mathrm{ for }\; x,y&\in D_{12}.\nonumber \\ \end{aligned}$$

We assume additionally that $r>0$ satisfies the following condition for sufficiently small $\varepsilon >0$

75 $$\begin{aligned} r>\frac{3a(\alpha _1^2+\alpha _2^2) +2\varepsilon }{(a-1)(\mu +\frac{1}{2}\alpha _2^2)-p-\frac{1}{2}\alpha _1^2}. \end{aligned}$$

Using the latter inequality we may similarly to the first part of the proof estimate the infinitesimal operator $A^*V(x,y)$ and obtain that $A^*V(x,y)<-\varepsilon$ for $x,y\notin K_1$, where $K_1\subset \mathbb {R}^2$ is the set bounded by the following straights

76 $$\begin{aligned} y&= z_0+\gamma -r, \quad y=\frac{(n+1)(z_0+\gamma )+x+r}{n}, \nonumber \\ x&= -(n+1)(z_0+\gamma ) +a(\gamma _1-\delta ) -r, \quad y=-(n+1)(z_0+\gamma ) +a(\gamma -\delta )-\gamma _2-r, \nonumber \\ y&= -(n+1)(z_0+\gamma )+x-\beta -r, \quad x=z_0+\gamma +\gamma _1 +r, \end{aligned}$$

where $$\beta =\frac{(n^2-2)\gamma -2n\delta -n\gamma _2}{n+1}-\gamma _1$$.

In the first part of the proof, under the assumption (58) we may construct the Khasminskií function in the more approachable form, we may obtain the following function

77 $$\begin{aligned} V(x,y)=\left( x-\log \left( \frac{\frac{\alpha _1^2}{2}+(2a-1)p}{2ap\alpha _1}\right) \right) ^4+ \left( y-x-\log \left( \frac{(\mu +\frac{\alpha _2^2}{2})(1+ke^y)}{2p(1+ke^y-a)}\right) \right) ^4\nonumber \\ \end{aligned}$$

and show that for the operator $A^*$ defined in (50) there exists $\varepsilon >0$ such that the following inequality holds

78 $$\begin{aligned} A^*V(x,y)<-\varepsilon \end{aligned}$$

for $x^2+y^2>K$ where $K>0$ is some sufficiently big but finite constant. The latter form of Khasminskií function is easier to generalise and use in the similar forms of the model (8).
